# Photothermal Performance of Lignin-Based Nanospheres and Their Applications in Water Surface Actuators

**DOI:** 10.3390/polym16070927

**Published:** 2024-03-28

**Authors:** Mingshan Wen, Hang Wang, Bole Ma, Fuquan Xiong

**Affiliations:** College of Materials Science and Engineering, Central South University of Forestry and Technology, No.498 at Shaoshan South Road, Changsha 410004, China; wenmingshan1031@163.com (M.W.); wanghang0110@163.com (H.W.); mabole0225@163.com (B.M.)

**Keywords:** ligninbased nanospheres, photothermal performance, hydrophobic coating, photothermaldriven actuator

## Abstract

In this study, the photothermal performance of lignin-based nanospheres was investigated. Subsequently, a photothermal actuator was prepared using lignin-based carbon nanospheres (LCNSs). The results demonstrated that LCNSs exhibited an impressive photothermal conversion efficiency of up to 83.8%. This extreme efficiency significantly surpasses that of lignin nanospheres (LNSs) and covalently stabilized LNSs (HT-LNSs). As a structural material, a hydrophobic coating was effectively engineered by LCNSs on the filter paper, achieving a water contact angle of 151.9° ± 4.6°, while maintaining excellent photothermal effects (with a temperature increment from room temperature to 138 °C in 2 s). When employing hydrophobic filter paper as the substrate for the photothermaldriven actuator, under the influence of a 1.0 W/cm^2^ power–density NIR laser, the material exhibited outstanding photothermal actuation, achieving speeds up to 16.4 mm/s. In addition, the direction of motion of the actuator can be adjusted in accordance with the location of the NIR light irradiation. This study offers valuable perspectives on the application of LNSs for highvalue applications and the development of innovative photothermal-driven actuators.

## 1. Introduction

Water surface actuators can convert external energy into kinetic energy, enabling controlled movement on various media surfaces such as oceans, rivers, and microfluidic surfaces [1,2]. Thus, they possess extensive potential for application in various domains, including scientific detection and monitoring of the environment. To date, multiple driving methods have been proposed for water surface actuators, including magnetic, electric, light, and chemical driving [2,3,4]. Compared to other driving methods, light driving does not require the discharge of chemical reagents into the water. This results in minimal impact on the aquatic environment and wirelessly controls the motion of the driving material. Based on the driving principle, light driving can generally be categorized into photothermal, photochemical, photoelectronic, and photomechanical driving [5,6,7,8,9]. Currently, most optically driven actuators are designed and manufactured based on the photothermal driving mode of the Marangoni effect [2,10,11,12,13,14]. This effect produces a temperature gradient that induces a variation in surface tension across the liquid, leading to the creation of microscale Marangoni currents. These currents are responsible for driving water surface actuators across the surface. To ensure swift photothermal actuation, two key criteria must be fulfilled: first, the actuator should exhibit an efficient photothermal response to rapidly establish a temperature gradient at the water’s surface; second, it is crucial to reduce the drag experienced during motion [10,15].

Photothermal materials harness the photothermal effect to produce gradients in temperature and surface tension, thereby imparting the driving force required for an actuator’s progression over liquid. Meanwhile, the surface hydrophilicity/hydrophobicity is usually controlled to minimize the resistance of the actuator during photothermal driving. When the actuator moves on the water’s surface, the hydrophobic surface can form a layer of air, which can significantly reduce the resistance of the actuator. In previous studies, polydimethylsiloxane (PDMS) with low surface energy was added to graphene oxide (GO) and carbon nanotubes (CNT) to obtain water surface actuators made by superhydrophobic, photothermal GO/PDMS and CNT/PDMS materials [8,16]. Additionally, superhydrophobic photothermal-driven water surface actuators were successfully obtained by chemically modifying photothermal materials such as polydopamine (PDA) and two-dimensional transition metal carbides/nitrides (MXenes) [11,15]. Nevertheless, existing photothermal-driven water surface actuators still face challenges such as slow response times, a low moving speed, and high-excitation power requirements [8,15]. Therefore, investigating how to prepare fast-moving photothermal-driven actuators with high photothermal conversion efficiency is a future research trend.

Lignin, being the second most plentiful renewable polymer in the natural world subsequent to cellulose, mainly comprises phenylpropane units connected via carbon–carbon and ether bonds. The complex structure of lignin makes it hard to achieve high-value utilization. Nanosizing lignin represents an innovative strategy for enhancing its valuable applications. Xiong et al. [17] prepared lignin nanospheres by dripping water into a tetrahydrofuran solution of lignin. They found that lignin molecules primarily aggregated through weak intermolecular forces in the solvent–antisolvent system, such as π-π bonds, hydrogen bonds, and electrostatic forces. The π-π stacking theoretically can promote non-radiative transitions of lignin nanomaterials and trigger photothermal conversion, making them applicable to photothermal materials [18]. Additionally, when lignin nanospheres (LNSs) are carbonized to form carbon nanospheres, the low bond strength between electrons can lead to lower energy requirements for electron transitions [19]. Theoretically, this allows for higher photothermal performance under a lower excitation energy. Therefore, applying the photothermal and small-size effects of lignin-based nanospheres to the construction of hydrophobic photothermal-driven actuators can effectively broaden the application scope of LNSs.

**Figure 1:**
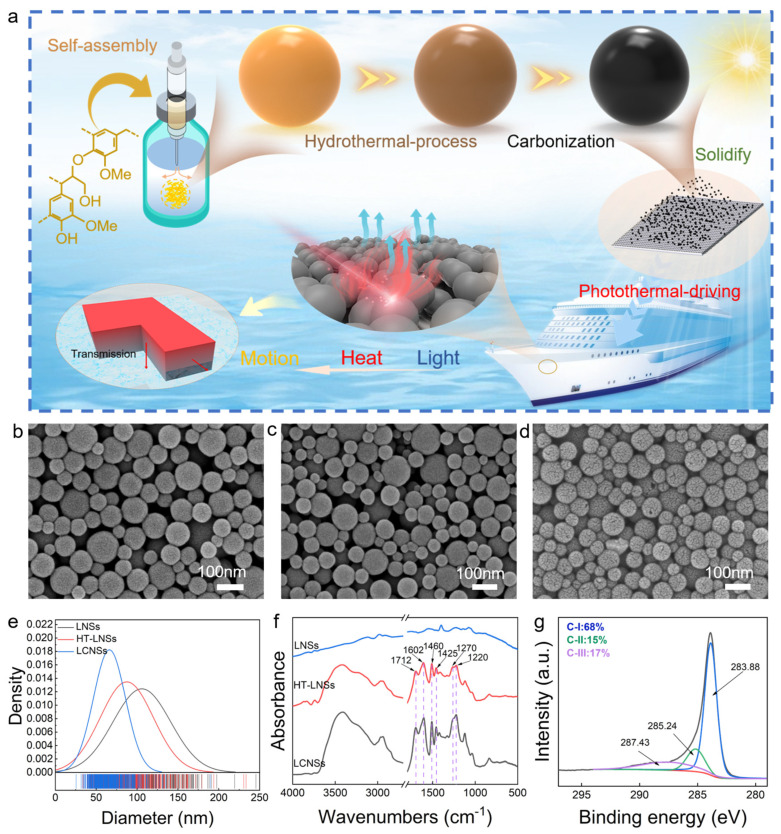
(**a**) Schematic illustration; (**b**–**d**) morphological examination of LNSs, HT-LNSs, and LCNSs through SEM; (**e**) the size distribution of LNSs, HT-LNSs, and LCNSs; (**f**) FTIR of LNSs, HT-LNSs, and LCNSs; and (**g**) XPS spectra with enhanced resolution for C 1s peaks of LCNSs.

In this study, using simple and fast preparation methods, the photothermal actuator was prepared by using lignin-based carbon nanospheres with high photothermal conversion efficiency. Fast and controllable motion was realized. LNSs (by the hydrophobic self-assembly of lignin molecules), covalently stabilized LNSs (HT-LNSs, by the hydrothermal treatment of self-assembled nanospheres), and lignin-based carbon nanospheres (LCNSs, by the carbonization of covalently stabilized nanospheres) were prepared, and the photothermal effect and stability of the three lignin-based nanospheres were investigated. Then, LCNSs with high photothermal conversion efficiency (η = 83.8%), which had higher photothermal conversion efficiency than those seen in other studies, were selected as structural materials to construct the photothermal hydrophobic coating on the filter-paper surface through a sedimentation method. In addition, their photothermal performance and water-surface driving properties were investigated (Figure 1a). The results revealed that, during photothermal driving, the actuator in this paper can achieve a driving speed of 16.4 mm/s under 1.0 W/cm^2^ near-infrared (NIR) laser, which is higher than other actuators developed by Cao et al. based on MXene and Wang et al. utilizing CNT, achieving velocities of 7.9 mm/s and 7.5 mm/s, respectively, at different power densities [8,15]. The direction in which the actuator moves can be steered by adjusting the direction of incident laser. The surface actuator prepared in this paper has achieved a breakthrough in high photothermal effect and fast motion speed, greatly broadening the applicability of photothermal hydrophobic filter paper in a fast photothermal driver, and offers a novel foundation for the development of biosensors, micro-robots, targeted drug-delivery systems, ecological conservation, and long-range sensing technologies.

## 2. Materials and Methods

### 2.1. Materials

Enzymatic hydrolysis lignin (EHL) was purchased from Hong Kong Laihe Biotech Co., Ltd., Hong Kong, China. As detailed in prior studies, the properties of the EHL under investigation have been delineated [20]. The hydroxyl group concentration was quantified at 1.87 mmol/g using 31P NMR analysis, into which 0.26 mmol/g for aliphatic-hydroxyl and 1.61 mmol/g for phenolic-hydroxyl were added. The number-average molecular weight and polydispersity index of EHL were assessed as being 1430 g/mol and 1.22, respectively, through the GPC analysis. The composition included 84.91% Klason lignin, exhibiting satisfactory solubility in tetrahydrofuran (THF). Prior to utilization, the EHL was dehydrated at 40 °C under vacuum conditions for a duration of 12 h and stored in a sealed, ground-glass-stoppered flask. 1H, 1H, 2H, 2H-perfluorooctyltriethoxysilane (F13-TMS) and γ-Valerolactone (GVL) were obtained from Shanghai Aladdin Biochemical Technology Co., Ltd., Shanghai, China. Analytical grade (AR) ethanol was purchased from Tianjin Hengxing Chemical Reagent Co., Ltd., Tianjin, China. Epoxy resin (EP, E51) and the curing agent (D230) were purchased from Jining Hua Kai resin Co., Ltd., Jining, China. All materials were used as received without further treatment.

### 2.2. Preparation of Lignin-Based Nanospheres

Enzymatic hydrolysis lignin was dissolved in a 1:1 (volume ratio) γ-valerolactone/deionized (DI) water binary solvent. After 30 min of ultrasonic dispersion, a saturated lignin solution, measuring 37.5 mg/mL, was prepared by centrifuging the mixture at 8000 rpm for a period of 20 min. The saturated solution was swiftly introduced into the DI water to achieve a γ-valerolactone volume fraction of 9%. Then, the solution obtained was moved into a dialysis sack designed to retain molecules larger than 1000 in molecular weight. and left for 48 h to eliminate any remaining γ-valerolactone, resulting in the production of LNSs. The dialyzed LNSs were hydrothermally treated at 160 °C for 12 h to obtain HT-LNSs. The HT-LNSs were carbonized in a tube furnace under nitrogen protection at 800 °C to obtain LCNSs.

### 2.3. Preparation of Carbon Nanospheres Hydrophobic Coating

LCNSs weighing 2.0, 4.0, and 6.0 mg were evenly distributed in a solution of ethanol (1.5 mL) using ultrasound-assisted dispersion. Subsequently, the dispersion was supplemented with F13-TMS (5 μL) and subjected to ultrasonic stirring at a speed of 800 rpm for a duration of 50 min. Following this, 9 mg of epoxy resin were added into the dispersion, which was stirred vigorously for half an hour. The, 3 mg of the curing agent were added and the dispersion was stirred for a further half hour to obtain a uniform mixture, which was then poured into a weighing bottle. Subsequently, a piece of filter paper, measuring 10 mm by 10 mm, was immersed in this mixture. Subsequently, the weighing bottle underwent heating at a temperature of 80 °C until complete evaporation of ethanol occurred. Additionally, curing was performed at a temperature of 100 °C for a duration of one hour, with the goal of obtaining hydrophobic filter paper. The 3 hydrophobic filter papers were designated as HP-2, HP-4, and HP-6 in sequential order. The side of the filter paper in contact with the bottom of the weighing flask was defined as the rear side, while the opposite side was referred to as the front side.

### 2.4. Characterization

The samples were examined using scanning electron microscopy (SEM, Zeiss sigma 300, Oberkochen, Germany) to observe their morphology. Fourier-transform infrared spectroscopy (FTIR, Vertex 70, Karlsruhe, Bruker) was employed to analyze the changes in functional groups of lignin-based nanospheres during hydrothermal treatment and carbonization. X-ray photoelectron spectroscopy (XPS, Axis Ultra, Kratos, Manchester, UK) was utilized for analyzing the surface chemical properties of lignin-based nanospheres. To determine the surface compositions of the hydrophobic coating, energy-dispersive X-ray spectroscopy (EDS, Zeiss sigma 300) was applied. Thermal-gravimetric analysis (TGA) was performed on the material in a nitrogen environment using a thermogravimeter (TG, Netzsch STA 449 F3, Bayern, Germany), and the sample was subjected to heating from 30 to 800 °C at a rate of 10 °C/min. The contact angle was determined at ambient temperature with the OCA15 CA system from Dataphysicals Instruments GmbH (Stuttgart, Germany). Contact-angle measurements were performed with a 4 μL water droplet. Each sample underwent five rounds of contact-angle testing and the results were averaged.

### 2.5. Photothermal Heating

LNSs, HT-LNSs, and LCNSs were mixed with ultrapure water to prepare solutions with concentrations of 10, 25, 50, and 100 μg/mL. Then, the solutions underwent irradiation employed an 808 nm NIR laser with a specified laser power density of 2.0 W/cm^2^ (duration = 10 min; Height = 5 cm) at room temperature, while pure water served as the blank control during the experiment. The temperature of the samples was monitored every 30 s during the process, and the fluctuations in temperature were documented by employing an infrared thermal imaging device. The photothermal conversion efficiency (η) can be calculated by [21]:

(1) $$\eta =\frac{hS{\Delta T}_{max}-Q_{dis}}{I\;(1-10^{-A})}$$

where *h* is the heat transfer coefficient; *S* is the surface area of the container; *hS* can be obtained by Equation (2); Δ*T*_max_ refers to the difference between the equilibrium and ambient temperatures; *Q*_dis_ refers to the amount of energy that is taken in by both the container and water, which is measured using a container with ultrapure water; *I* refers to the incident laser power density, which is measured at 2.0 W/cm^2^; and *A* is the absorbance value of the sample solution at a wavelength of 808 nm.

(2) $$hS=\frac{mC}{\tau_{s}}$$

where *m* is the mass of the solution, C is the specific heat of water, and $\tau_{s}$ is the slope of the fitted line.

### 2.6. Photothermal Stability

LNS, HT-LNS, and LCNS solution samples were subjected to an 808 nm NIR laser (concentration = 100 μg/mL and laser power density = 2.0 W/cm^2^). The laser was turned off after 600 s, allowing the samples to cool naturally to room temperature. This process was repeated three times for each sample group. The sample temperature was recorded every 30 s using an infrared thermal imager. For the hydrophobic filter papers, the laser power density was adjusted to a level of 1.0 W/cm^2^, followed by an irradiation duration of 30 s. This measurement was repeated for a total of five consecutive on-and-off cycles.

### 2.7. Photothermal Driving Performance

The 808 nm NIR laser was employed to assess both the photothermal efficiency and photothermal driving performance of HP-2, HP-4, and HP-6. By adjusting the NIR laser power density and the incident laser irradiation position (the distance is controlled at 10 cm), the motion speed and trajectory of the hydrophobic filter papers were recorded.

## 3. Results and Discussion

### 3.1. Morphology and Chemical Structural Characteristics of Lignin-Based Nanospheres

The SEM images of the LNSs, HT-LNSs, and LCNSs are depicted in Figure 1b–d. As shown in Figure 1b, the LNSs had a well-defined spherical structure after self-assembly (Figure 1b). The HT-LNSs maintained a solid spherical morphology after the hydrothermal process (Figure 1c), but the average particle size showed a moderate reduction (Figure 1e). This might have resulted from the interaction of lignin molecules through a condensation reaction during the hydrothermal process, leading to a decrease in the particle size of the LNSs [22]. After carbonization, the LCNSs still maintained a spherical structure but showed minor surface cracks (Figure 1d). This was possibly due to the further contraction of nanospheres during carbonization [23]. This also contributed to a further reduction in the average particle size to 66.2 nm.

XPS and FTIR analysis were performed to investigate the chemical structure changes of lignin-based nanospheres during the hydrothermal and carbonization processes. As shown in Figure 1f, LNSs displayed vibrational peaks at 3400 cm^−1^ (linked to hydroxyl-group stretching), 2930 cm^−1^ (associated with methyl- and methylene-group stretching), 2840, 1460, and 1425 cm^−1^ (reflective of methoxy-group stretching), and 1712 and 1329 cm^−1^ (indicating carbonyl group stretching modes). 1602 cm^−1^ (associated with the stretching vibration of the aromatic skeleton), as well as at 1270 cm^−1^ (attributed to methoxy-group stretching) and at 1220 cm^−1^ (related to ether-bond stretching). Compared to the LNSs, the absorption peak of HT-LNSs at 1602 cm^−1^ was basically unchanged, suggesting minimal changes in the aromatic ring skeleton during the heat-stabilization process. However, the absorption peaks at 3400, 2930, 2840, 1460, 1425, and 1220 cm^−1^ gradually weakened. This phenomenon can be attributed to the breakdown of chemical linkages possessing lesser energy required for dissociation, like those found in methoxy and β-O-4 aryl ether connections. In comparison to the LNSs and HT-LNSs, the disappearance of peaks at 3400 and 2930 cm^−1^ was observed in the LCNSs, accompanied by a significant reduction in intensity for other characteristic peaks (1712, 1602, 1460, and 1220 cm^−1^). These findings indicate the complete decomposition of lignin during the carbonization process [24].

As shown in Figure S1, C and O could be detected in all three samples, corresponding to peaks at 285 and 533 eV in the spectra, respectively. Compared with the LNSs, the carbon and oxygen contents of the HT-LNSs decreased from 76.22 to 74.69 at.% and increased from 21.88 to 23.79 at.%, respectively. This was mainly because the self-condensation of lignin molecules was more significant than the cleavage of chemical bonds [25]. After carbonization, the carbon content of the LCNSs increased to 94.58 at.% while its oxygen content decreased to 4.42 at.%. This suggests that non-carbon atoms in the LCNSs were gradually eliminated during carbonization.

Detailed C 1s and O 1s spectral data of the lignin-based nanospheres were obtained to clarify the surface functional groups (Figure 1g and Figure S2). In the C 1s spectra (Figure S2a), three dependent characteristics peaks were observed, corresponding to C-C or C-H (C-I, 284.67 eV), C-OH (C-II, 286.23 eV), and O-C=O (C-III, 287.77 eV) [26]. In the O1s spectra (Figure S2d), two dependent characteristics peaks were observed at 531.24 and 532.96 eV, corresponding to Ph=O, C=O or Ph-C=O (O-I), and Ph-OH or C-O-C (O-II), respectively [27]. After hydrothermal covalent stabilization, the content of C-I decreased from 58 to 55 at.%. This can be ascribed to the partial detachment of carbon-containing groups by the demethylation reaction during hydrothermal treatment [22]. Moreover, the content of O-II decreased from 25 to 21 at.% after hydrothermal treatment, while the contents of carboxyl and carbonyl (C-III, O-I) increased slightly, due to the uniform cleavage and dehydration reaction of chemical bonds with lower bond-dissociation energies [24]. After further carbonization, the high content of C-I in the LCNSs (Figure 1g) and low peak intensity of O1s in LCNSs (Figure S2f) revealed that a substantial amount of oxygen-containing groups was removed, resulting in a higher degree of carbonization in the LCNSs.

### 3.2. Photothermal Performance of Lignin-Based Nanospheres

**Figure 2:**
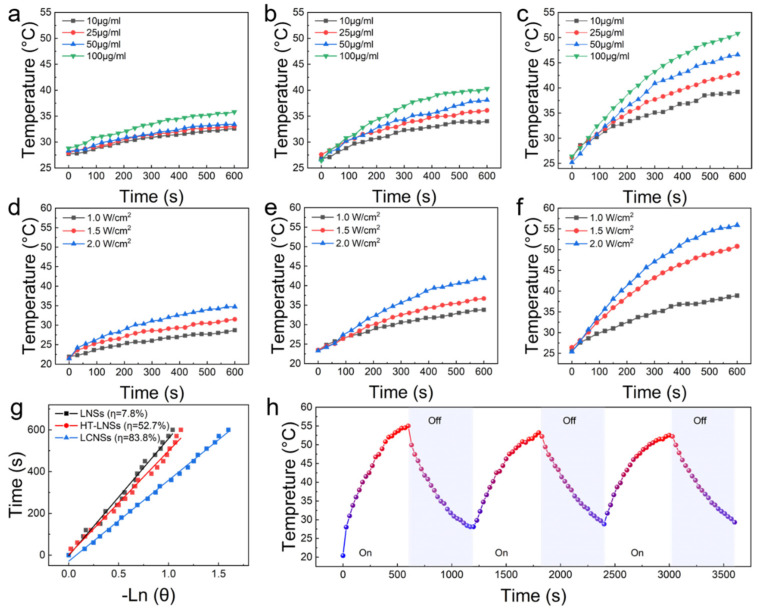
(**a**–**c**) Temperatures elevation of LNS, HT-LNS, and LCNS aqueous suspensions (NIR light power = 1.5 W/cm^2^) with different concentrations as a function of irradiation time; (**d**–**f**) temperatures elevation of LNS, HT-LNS, and LCNS aqueous suspensions (100 μg/mL) with different NIR laser power densities as a function of irradiation time; (**g**) linear time data versus −ln*θ* obtained from the cooling period; and (**h**) temperature monitoring of LCNS aqueous suspension (100 μg/mL) during three successive cycles of an on-and-off laser.

The conjugated structure of lignin nanoparticles facilitates the efficient movement of electrons from lower- to higher-energy orbitals. The light energy absorbed is predominantly emitted through non-radiative transitions, leading to an increase in the material temperature [28]. Under the irradiation of an 808 nm NIR laser (1.5 W/cm^2^), the temperatures increased accordingly with increases in the concentrations of the LNS, HT-LNS, and LCNS solutions (Figure 2a–c). When the solution concentration was 100 μg/mL, the temperatures of the LNS and HT-LNS solutions increased by 7.0 and 13.9 °C within 600 s, respectively. After carbonization, the temperature of the LCNS solution increased by 24.4 °C (Figure 2c). This might be because high-temperature carbonization induced the formation of numerous micro-defects inside the carbon layer. These defect sites exhibited high activity, and the excited electrons underwent non-radiative recombination around the defect sites, which further enhanced the photothermal performance of the lignin-based carbon spheres [29,30].

At a solution concentration of 100 μg/mL, the impact of light power on the solution temperature was further investigated (Figure 2d–f). With increasing power, the temperature of pure water remained almost unchanged, even at a laser power density of 2.0 W/cm^2^ (Figure S3), while the maximum temperatures of the LNSs, HT-LNSs, and LCNSs increased. It is surprising that the LCNSs showed a faster temperature increment rate and a higher final destination temperature. Compared with the LNSs and HT-LNSs, the LCNSs had a better photothermal performance and the photothermal performance could be precisely controlled by adjusting the NIR laser power.

Considering the reuse of lignin-based nanospheres as photothermal materials, the photothermal cyclic stability of the LNSs, HT-LNSs, and LCNSs was tested (Figure 2h). In three consecutive cyclic tests, the heating and cooling curves of LNSs, HT-LNSs, and LCNSs showed similar trends, and the maximum temperature remained basically the same, demonstrating good photothermal cyclic stability. A complete heating and cooling conversion process of the LNSs, HT-LNSs, and LCNSs was fitted (Figure S4a,c,e) based on Equations (1) and (2). Herein, the mass of pure water (*m*) was 2 g, and the specific heat of the water was 4.2 J/(g·K). According to the slopes of the fitted lines for the respective cooling times and the negative natural logarithms of the driving-force temperatures, *τ*_s_ values of the LNSs, HT-LNSs, and LCNSs were 560, 497, and 388 ms, respectively. By substituting these values into Equations (1) and (2), the photothermal conversion efficiencies (*η*) of LNS, HT-LNS, and LCNS were calculated to be 7.8%, 52.7%, and 83.8%, respectively. Compared with the carbon-based photothermal materials reported by previous researchers, such as hollow carbon nanospheres at 35.7% [31], solid carbon spheres at 54.2% [32], mesoporous nanospheres at 35.8% [33], and reduced graphene oxide at 25.8% [34], the photothermal conversion efficiency of the LCPs examined here shows a significant enhancement. After covalent stabilization, the interaction between the lignin molecules promoted π-π stacking, thereby enhancing photothermal conversion. Moreover, the LCNSs exhibited a more pronounced photothermal effect. This further demonstrates the significance of carbonization in improving the photothermal performance of lignin-based nanospheres.

### 3.3. Characterization and Photothermal Performance of LCNS Hydrophobic Coating

**Figure 3:**
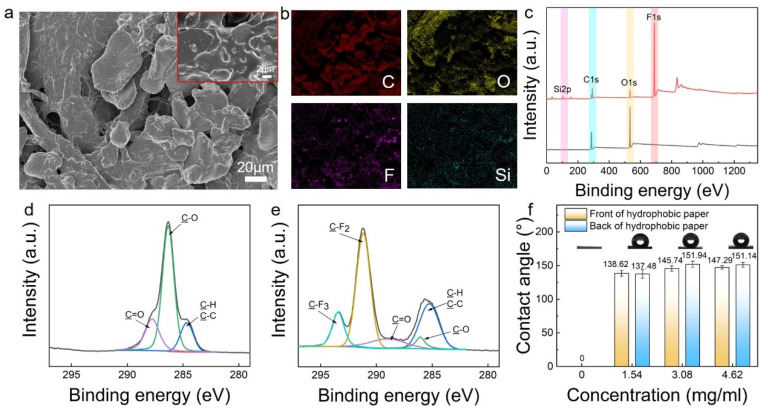
(**a**) SEM analysis of the morphology of the rear side of the hydrophobic filter paper; (**b**) EDS mapping of the rear side of the hydrophobic filter paper; (**c**) wide-scan XPS spectra of the filter paper and hydrophobic filter paper; (**d**) high-resolution XPS spectra of C 1s peaks of the filter paper; (**e**) high-resolution XPS spectra of C 1s peaks of the hydrophobic filter paper; and (**f**) contact angle of the filter paper, and both sides of HP-2, HP-4 and HP-6.

In view of the excellent photothermal and small-size effect of the LCNSs, the derived hydrophobic photothermal coating was constructed on the filter-paper surface. SEM images revealed (Figure 3a) that the LCNSs were anchored to the matrix surface after bonding with epoxy resin, forming micro/nano protrusions. According to the Cassie-Baxter model, this layered micro/nano-structure can improve hydrophobicity [35,36]. Additionally, the introduction of fluorinated organic molecules can further reduce the surface energy of the coating. Successful modification of F13-TMS in the coating was demonstrated by EDS and XPS. The EDS analysis revealed the presence of C, N, O, F and Si in the coating. The detection of F from F13-TMS (Figure S5a,g) indicated the successful hydrophobic modification of the LCNSs. Furthermore, the EDS mapping demonstrated a homogeneous distribution of C, O, F, and Si on the hydrophobic filter paper (Figure 3b and Figure S5). The XPS spectra of the hydrophobic coating also revealed (Figure 3c) the presence of F 1s and Si 2p peaks, in addition to the C 1s and O 1s peaks. It is worth noting that the high-resolution XPS spectra (Figure 3e) exhibited distinct C-F peaks at 291.2 and 293.4 eV, providing further evidence for the successful introduction of F13-TMS.

Figure S6 shows the thermogravimetric analysis of the filter paper and the hydrophobic coated filter paper. The loss ratio of the ordinary filter paper reached 82.91 wt.%, and the loss ratio of the HP-2 was 79.45 wt.%, Since the carbon nanosphere is carbonized in a nitrogen atmosphere at 800 °C, the loss of the carbon nanosphere in thermogravimetric analysis can be ignored. The loss ratio of HP-4 and HP-6 is lower but almost equal (76.39 wt.% and 76.14 wt.%, respectively), which led to the speculation that the introduction of more carbon nanospheres did not increase the amount of nanospheres settling on the filter-paper surface.

Utilizing a contact-angle measurement device, the water contact angles on each side of the hydrophobic filter paper were measured, aiming to assess the wetting characteristics of the hydrophobic coating. As shown in Figure 3f, the filter paper was hydrophilic (the water contact angle was 0°), while HP-2, HP-4, and HP-6 were all hydrophobic. The contact angles of HP-4 and HP-6 were nearly identical. This confirmed the results of the thermogravimetric analysis. Notably, the mean contact angles of HP-4 and HP-6 were close to those of the superhydrophobic material. Nevertheless, the average contact angles on the front side of the hydrophobic filter paper was slightly lower than that on the rear side (Figure 3f). According to Figure S5b, this might be because excessive settling of the epoxy resin on the front side caused whole-piece adhesion, reducing the surface micro/nano roughness. There are many air gaps on the rough surface, the water droplets cannot penetrate, and it is easy to roll away, so the high roughness on the rear side obtains higher contact angles [37,38].

**Figure 4:**
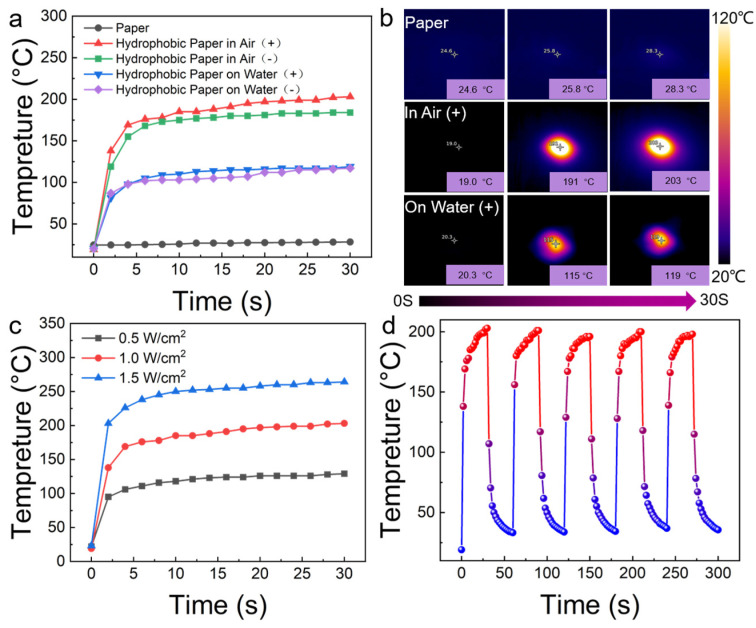
(**a**) Temperature elevation of the filter paper and both sides of HP-2, HP-4 and HP-6 in air and on the water’s surface under 808 nm NIR laser (1.0 W/cm^2^) as the duration of exposure to radiation increases; (**b**) the IR images of the filter paper and HP-4 in air and on the water’s surface; (**c**) the temperatures elevation of the front side of HP-4 with different NIR laser power densities as the duration of exposure to radiation increases; and (**d**) temperature monitoring of the front side of the hydrophobic filter paper during five consecutive laser cycles with alternating on-and-off periods.

HP-4 was selected to further evaluate the photothermal performance. As observed, the NIR laser irradiation (at 1.0 W/cm^2^) led to minimal variations in the surface temperature of the ordinary filter paper. After hydrophobic modification, the temperature on the front side of the filter paper rapidly increased to 138 °C within 2 s, finally reaching approximately 203 °C, which was higher than that of the rear side (184 °C). The significant and rapid temperature change demonstrates the good photothermal response of the hydrophobic filter paper (Figure 4a). As shown in Figure 4c, even with a reduced laser power intensity of 0.5 W/cm^2^, the equilibrium temperature on the front side of the hydrophobic filter paper reached 129 °C. When the power density was increased to 1.5 W/cm^2^, the equilibrium temperature reached up to 264 °C. Then, the photothermal cyclic stability of the hydrophobic filter paper was investigated through five cycles of irradiation by the NIR laser (1.0 W/cm^2^) (Figure 4d and Figure S7). The temperature variation trends on both sides of the hydrophobic filter paper remained almost consistent, confirming good photothermal cyclic stability.

The photothermal performance of the hydrophobic filter paper as a photothermal-driven actuator on the water surface was further studied (Figure 4a,b). Exposed to the near-infrared laser illumination at 1.0 W/cm^2^, when the hydrophobic filter paper was placed with the front side facing up on the water surface, the water surface temperature could reach 119 °C, which was slightly higher than that of the rear side (117 °C). What is more, temperature gradient radiation appeared around the irradiation point. This suggests that the heat generated by the hydrophobic filter paper was efficiently transferred to the surrounding water surface. The generation of a temperature gradient is necessary for the movement of the photothermal-driven actuator, based on the Marangoni effect. With efficient photothermal conversion capability and good hydrophobicity, the hydrophobic filter paper serves as an ideal material for a water surface photothermal-driven actuator.

### 3.4. Driving Performance of the Photothermal-Driven Actuator

According to the hydrophobicity and photothermal performance on both sides of the hydrophobic filter paper, a photothermal-driven actuator was constructed with the front side of the hydrophobic filter paper for photothermal conversion and the rear side for reducing the resistance to water surface movement. Controllable motion of the photothermal-driven actuator can be accomplished by regulating both the position and power of the incident laser irradiation. Photothermal driving primarily relies on the surface tension of the liquid. When the actuator was positioned on top of the water, there was an equal distribution of surface tensions in all directions. When the NIR laser was irradiated on one side, the actuator surface transferred heat to the liquid surface through photothermal conversion. Based on the Marangoni effect, the surface tension of liquids with higher temperatures is smaller, and the liquid will move towards the adjacent liquid that exhibits higher surface tension, thereby driving the actuator to move in the opposite direction [11,39]. Additionally, due to the surface hydrophobicity, the actuator formed a thin air layer on the water surface, enabling it to float on the water surface, significantly reducing motion resistance [40]. As shown in Figure S8, the side irradiated with the NIR laser showed an increased temperature (T_1_), a reduced surface tension coefficient (γ_1_), and reduced surface tension (F_1_). Hence,

T_1_ > T_2_; therefore, γ_1_ < γ_2_, F_1_ < F_2_ (3)

According to the momentum theorem,

FΔt = mΔv,(4)

where m denotes the mass of the photothermal-driven actuator, and t denotes the time. As observed, with an increase in the resultant force (F), the movement speed (v) of the hydrophobic coated filter paper accelerates.

**Figure 5:**
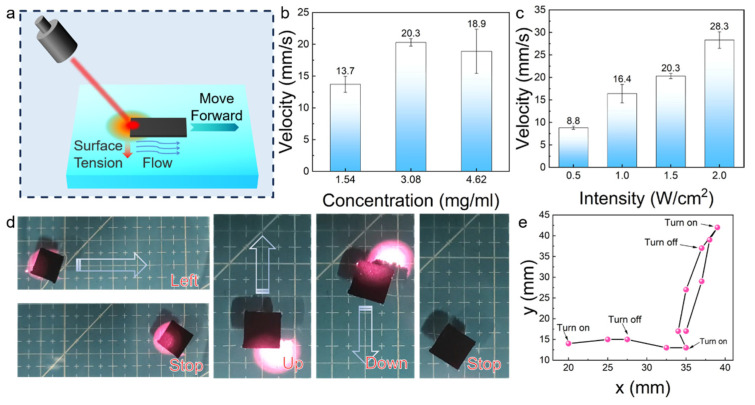
(**a**) Schematic description of the photothermal-driven actuator movement induced by the Marangoni effect; (**b**) velocity histogram of HP-2, HP-4 and HP-6 under 808 nm NIR light (2.0 W/cm^2^); (**c**) velocity histogram of HP-4 under different NIR laser power densities; (**d**) optical motion images of HP-4; and (**e**) motion track of HP-4 under 808 nm NIR light (1.5 W/cm^2^).

When the NIR laser stopped irradiation, the heated side no longer generated heat, and the temperature gradually decreased to room temperature. The surface tensions on both sides of the liquid returned to an equal state, causing the actuator to stop moving. Therefore, the movement state can be controlled using the switch of the NIR laser (Figure 5). As shown in Figure 5d, the NIR laser was irradiated on the left side of the photothermal-driven actuator to control its movement. The average velocity of the hydrophobic filter paper can be determined by analyzing the relationship between time and displacement (Figure 5b). The HP-2 exhibited a noticeably lower movement speed than HP-4 and HP-6, due to low photothermal performance and hydrophobicity. In addition, the HP-6 did not further enhance the movement speed.

The HP-4 was chosen to further study the driving performance of the actuator. The movement speed of the photothermal-driven actuator on the water surface was controlled by changing the power density of the NIR laser. As the laser power gradually increased (Figure 5c), the movement speed of the actuator increased from 8.8 mm/s (0.5 W/cm^2^) to 28.3 mm/s (2.0 W/cm^2^). The movement path of the actuator on the water surface is depicted in Figure 5e. The actuator was induced to move in the opposite direction by laser irradiation applied on one side, and adjusting the position of irradiation enabled reciprocating motion. This further demonstrates that the actuator made with the hydrophobic filter paper has excellent wireless driving controllability on the water surface. Table 1 presents a comparative overview of the actuator performance from this study with other recent findings in the field. At equivalent laser power densities, the lignin-based actuators exhibit a higher average velocity than those previously reported. Across various power densities, the developed actuators also show a propensity for quicker velocity. Specifically, the actuator detailed herein reaches a velocity of 16.4 mm/s at a modest laser power density of 1.0 W/cm^2^, which is notably higher than the velocities documented for actuators at more intense power levels. This suggests that the hydrophobic filter paper used in this study offers distinct advantages as an actuator material when compared to other materials reported in the literature. The enhanced velocity is attributed to the hydrophobic photothermal nature of the hydrophobic filter paper. The photothermal conversion of actuators must be efficiently realized within seconds; therefore, the temperature increases of the actuator under 1.0 W/cm^2^ irradiation for 5 s was analyzed. The actuator in this paper achieved efficient photothermal conversion, with a temperature rise of 84.7 °C in just 5 s, which is superior to that of other actuators reported in the literature. Therefore, using the hydrophobic filter paper made of lignin-based carbon spheres as photothermal hydrophobic materials for photothermal-driven actuators has significant advantages and application potential.

## 4. Conclusions

The lignin-based nanospheres were prepared by combining self-assembly, hydrothermal covalent stabilization, and carbonization methods. Under a concentration of 100 μg/mL and an NIR laser power density of 2.0 W/cm^2^, LNSs, HT-LNSs, and LCNSs exhibited excellent photothermal performance, which reached temperatures of 34.7, 41.9, and 55.9 °C, respectively. Photothermal cyclic stability testing revealed excellent photothermal stability and remarkable conversion efficiency (7.8%, 52.7%, and 83.8%, respectively) for the as-prepared lignin-based nanospheres. The LCNS were used in the fabrication of a hydrophobic coating on filter-paper surfaces. The average water CA on the front sides of HP-2, HP-4, and HP-6 were 137.5° ± 6.4°, 151.9° ± 4.6°, and 151.1° ± 3.6°, respectively, while those on the rear side were 138.6° ± 4.3°, 145.7° ± 4.1°, and 147.3° ± 2.7°. The maximum temperatures of the photothermal-driven actuator constructed by HP-4 on both sides reached 203 °C and 184 °C in the air and 119 and 117 °C on the water, respectively. The movement speed reached 16.4 mm/s under the NIR laser irradiation (1.0 W/cm^2^), beyond what has been reported. In addition, the movement direction can be adjusted by changing the position where incident laser was applied. This study provides valuable insights into potential applications of LNSs in fields such as photothermal-driven robotics and water-surface transportation.

## Figures and Tables

**Table 1:** Comparison of photothermal driving performance between HP-4 and other photothermal actuators in the literature.

Intensity of the Irradiation [W/cm^2^]	Contact Angle [°]	Velocity of the Actuator [mm/s]	Reference
1.0	——	2.4	Song et al. [41]
0.9	153.6	4.6	Wang et al. [42]
1.0	163.1	7.9	Cao et al. [15]
1.5	157.4	8.9	Wu et al. [4]
3.4	161.8	7.5	Wang et al. [8]
1.5	151	20.3	This work

## Data Availability

Data are contained within the article and Supplementary Materials.

## Supplementary Materials

The following supporting information can be downloaded at: https://www.mdpi.com/article/10.3390/polym16070927/s1: Figure S1: Wide-scan XPS spectra of LNSs, HT-LNSs and LCNSs. Figure S2: High-resolution (a–c) C 1s and (d–f) O 1s XPS spectra of LNSs, HT-LNSs and LCNSs, respectively. Figure S3: Temperatures elevation of deionized water under 808 nm NIR light (2.0 W/cm^2^) as a function of irradiation time. Figure S4: (a,c,e) The photothermal response of LNSs, HT-LNSs and LCNSs aqueous solution (100 μg/mL) for 600 s under 808 nm NIR laser (2.0 W/cm^2^) and then the laser was shut off. (b,d,f) Linear time data versus -ln*θ* obtained from the cooling period of Figure S4 (a,c,e), respectively. Figure S5: (a,g) EDS spectra and (b–f,h–l) EDS mapping of the front side and the rear side of the hydrophobic filter paper. Figure S6: Thermogravimetric analysis diagram of filter paper and hydrophobic coated filter paper. Figure S7: Temperature monitoring of the front side of the hydrophobic filter paper during successive five cycles of an on-and-off laser. Figure S8: Motion mechanism of photothermal driving based on the Marangoni effect.
