# Supplementary material for: Photothermal Performance of Lignin-Based Nanospheres and Their Applications in Water Surface Actuators
Source: Polymers (Basel). 2024 Mar 28;16(7):927. doi: 10.3390/polym16070927 (PMC11013333; doi:10.3390/polym16070927)
Supplement: Supplementary file 1 [file polymers-16-00927-s001.zip › polymers-2904565-supplementary.pdf]

Supporting Information

Photothermal Performance of Lignin-Based Nanospheres and Their Applications in Water Surface Actuators

Mingshan Wen, Hang Wang, Bole Ma and Fuquan Xiong *

College of Materials Science and Engineering, Central South University of Forestry and Technology, No.498 at Shaoshan South Road, Changsha 410004, China; wenmingshan1031@163.com (M.W.); wanghang0110@163.com (H.W.); mabole0225@163.com (B.M.)

* Correspondence: xiongfuquan@126.com

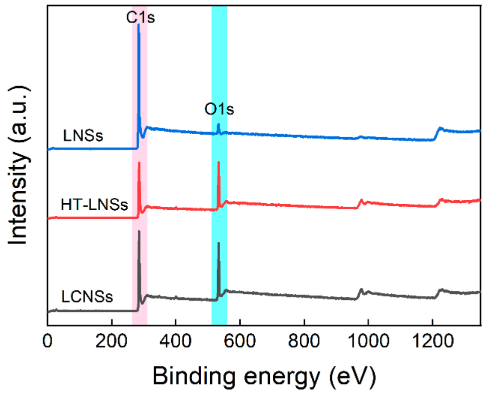

Figure S1. Wide-scan XPS spectra of LNSs, HT-LNSs and LCNSs.

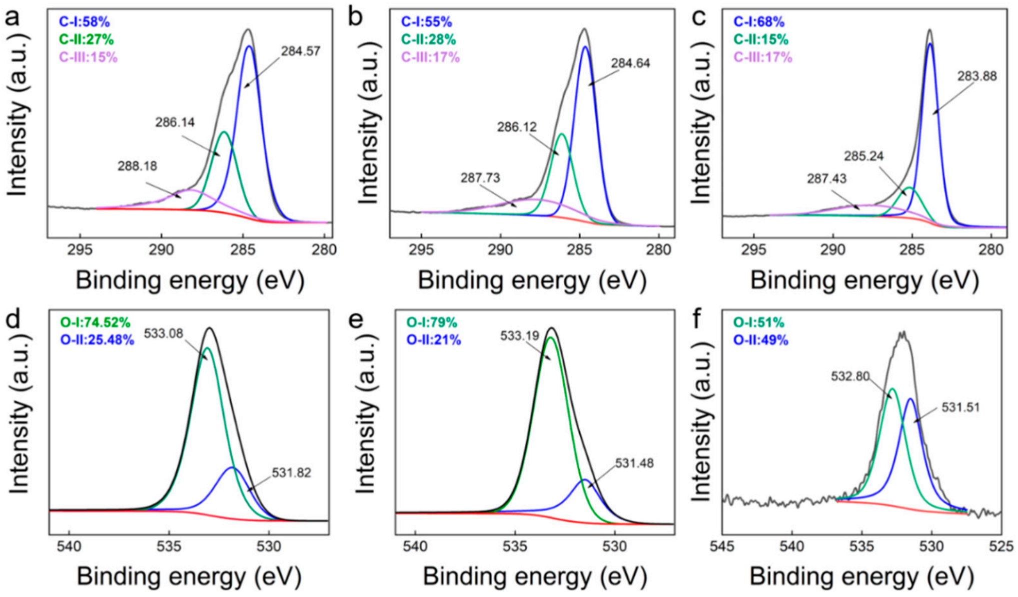

Figure S2. High-resolution (**a-c**) C 1s and (**d-f**) O 1s XPS spectra of LNSs, HT-LNSs and LCNSs, respectively.

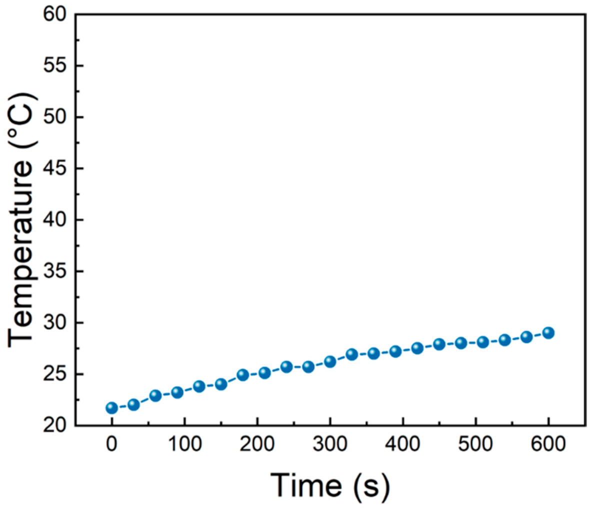

Figure S3. Temperatures elevation of deionized water under 808 nm NIR light (2.0 W/cm²) as a function of irradiation time.

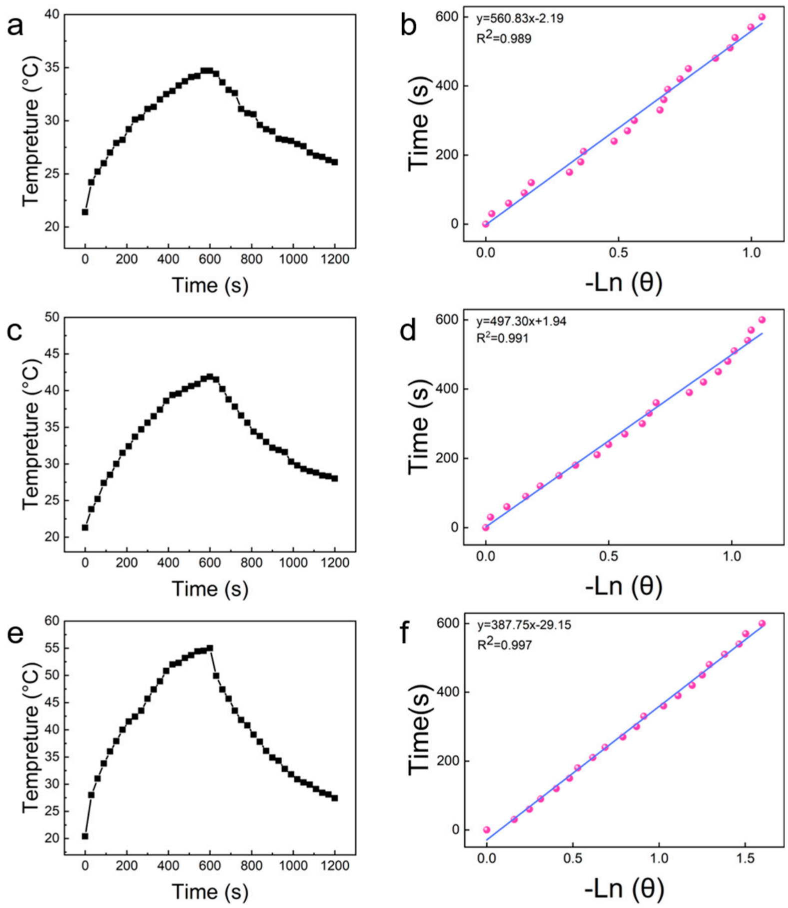

Figure S4. (**a**, **c**, **e**) The photothermal response of LNSs, HT-LNSs and LCNSs aqueous solution (100 μg/mL) for 600 s under 808 nm NIR laser (2.0 W/cm²) and then the laser was shut off. (**b**, **d**, **f**) Linear time data versus −lnθ obtained from the cooling period of Figure S4 (**a**, **c**, **e**), respectively.

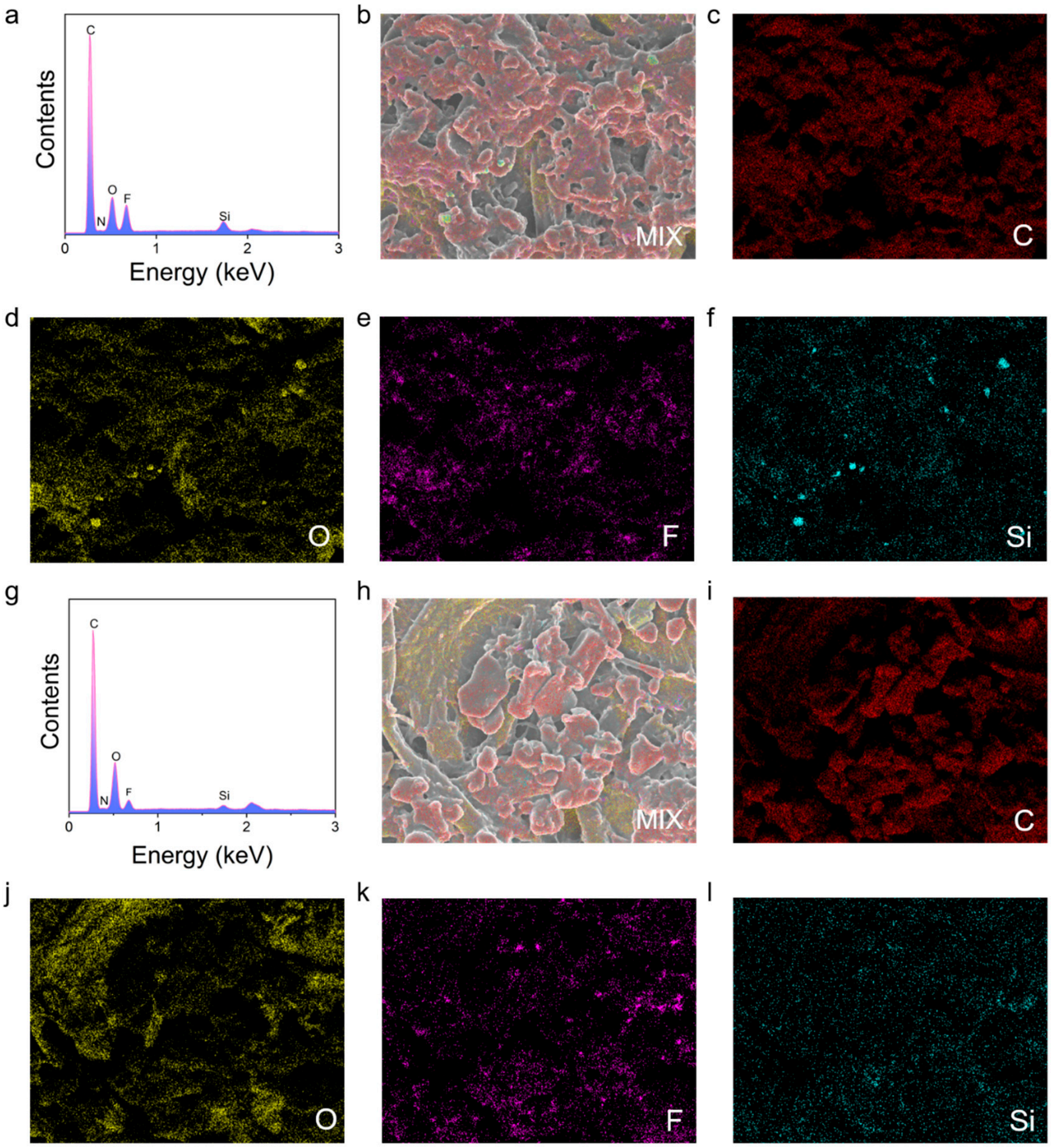

Figure S5. (**a**, **g**) EDS spectra and (**b-f**, **h-l**) EDS mapping of the front side and the rear side of the hydrophobic filter paper.

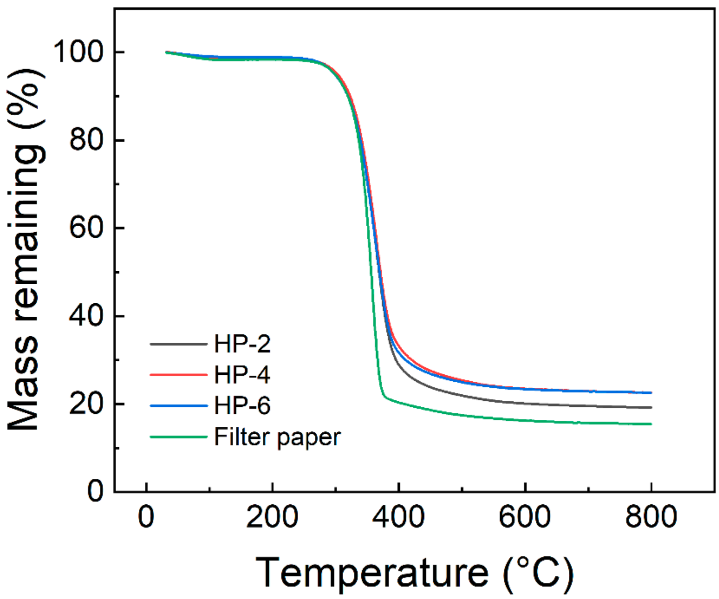

Figure S6. Thermogravimetric analysis diagram of filter paper and hydrophobic coated filter paper.

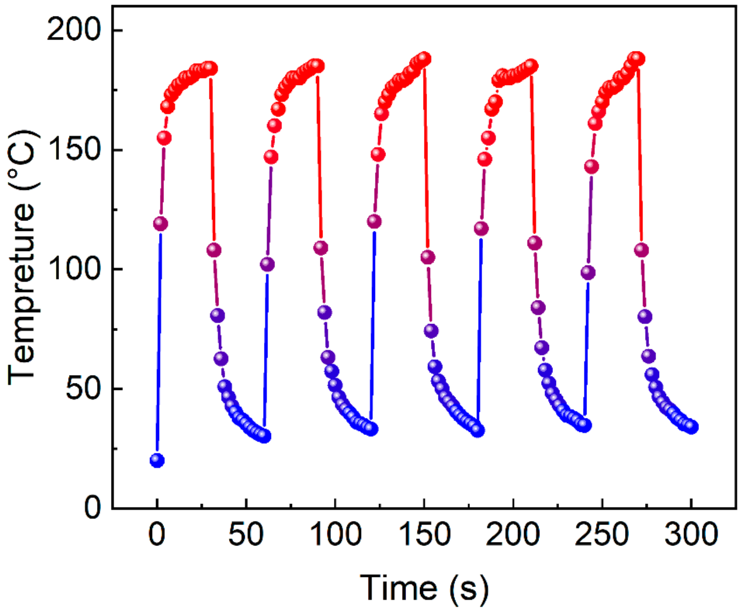

Figure S7. Temperature monitoring of the front side of the hydrophobic filter paper during successive five cycles of an on-and-off laser.

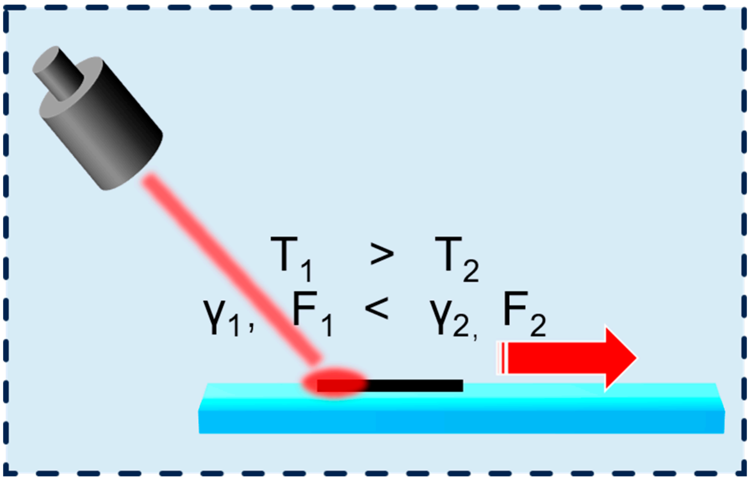

Figure S8. Motion mechanism of photothermal driving based on the Marangoni effect.
